# Whole-Genome Sequence of *Mycobacterium kyorinense*

**DOI:** 10.1128/genomeA.01062-14

**Published:** 2014-10-16

**Authors:** Kouki Ohtsuka, Hiroaki Ohnishi, Eriko Nozaki, Jesus Pais Ramos, Enrico Tortoli, Shota Yonetani, Satsuki Matsushima, Yoshitaka Tateishi, Sohkichi Matsumoto, Takashi Watanabe

**Affiliations:** a Department of Laboratory Medicine, Kyorin University School of Medicine, Tokyo, Japan; b National Reference Laboratory for Tuberculosis, Centro de Referência Professor Hélio Fraga, Escola Nacional de Saúde Pública, Fiocruz, Rio de Janeiro, Brazil; c Emerging Bacterial Pathogens Unit, San Raffaele Scientific Institute, Milan, Italy; d Department of Bacteriology, Niigata University Graduate School of Medicine, Niigata, Japan

## Abstract

We report here the first draft genome sequence of *Mycobacterium kyorinense*, which was described in 2009 and exhibits significant pathogenicity to humans.

## GENOME ANNOUNCEMENT

*Mycobacterium kyorinense* is a slow-growing mycobacterium that was first described in 2009 (1). *M. kyorinense* is closely related to *M. celatum*, *M. branderi*, and *M. fragae*, and exhibits significant pathogenicity for humans, causing pneumonia, lymphadenitis, and arthritis (2–4). Antimicrobial susceptibility tests demonstrated that *M. kyorinense* is generally resistant to rifampin, isoniazid, and ethambutol (4). Further investigation is needed to clarify the genomics, biology, epidemiology, and pathogenicity of this species.

We sequenced the genomic DNA of the *M. kyorinense* type strain KUM060204^T^ on an Ion PGM system (Life Technologies) and assembled the reads using CLC Genomics Workbench 7.0. A total of 4,133,490 reads were generated, with an average read length of 203 bp, yielding a total sequence of 837,657,777 bp.

The assembled sequences of KUM060204^T^ comprised 453 contigs, with a combined length of 5,302,980 bp, with a G+C ratio of 66.9%. The average cover depth was 50×, the *N*_50_ contig size was 53,523, the average contig was 11,706 bp long, and the longest contig was 137,319 bp.

Genome annotation was performed using the RAST prokaryotic genome annotation server (http://www.nmpdr.org/FIG/wiki/view.cgi/Main/RAST). RAST predicted 5,405 putative open reading frames, including 5,351 coding sequences and 54 RNAs (46 tRNAs and 8 rRNAs). RAST functional analysis of the predicted protein-coding genes showed 78 genes involved in cell walls and capsules, 64 in membrane transport, 206 in protein metabolism, 93 in DNA metabolism, 141 in virulence and defense, 135 in respiration, 331 in fatty acids, lipids, and isoprenoids, 395 in cofactors, vitamins, prosthetic groups, and pigments, and 356 in amino acids and derivatives.

To explore the molecular mechanism underlying the resistance of *M. kyorinense* to anti-tuberculosis drugs, we selected several genes known to be responsible for resistance to rifampin (*rpoB*), ethambutol (*embB*), and isoniazid (*inhA*, *katG*, and *ahpC*). The sequences of these genes in *M. kyorinense* were compared with those in *M. tuberculosis* H37Rv to clarify whether they contain specific mutations associated with resistance to anti-tuberculosis drugs in *M. tuberculosis*. Analysis of the *rpoB* gene confirmed our previous finding that KUM060204^T^ harbors a Ser531Asp amino acid substitution, the most frequent mutation in rifampin-resistant *M. tuberculosis* (4, 5). In contrast, we did not detect a substitution at Met306 of *embB*, the major mutation in ethambutol-resistant *M. tuberculosis* (6). Nor did we find a Ser315Thr substitution of *katG*, a mutation in the regulatory region (nucleotides [nt] −48, −51, −54, −81, and −88) of *ahpC*, a Ser94Ala substitution in the *inhA* gene or a mutation in the regulatory region (nt −15 and −17) of *inhA*, which are common mutations in isoniazid-resistant *M. tuberculosis* (6). These results suggested that the mechanism underlying drug resistance in *M. kyorinense* is significantly different from that in *M. tuberculosis.*

In conclusion, we report the genome sequence of KUM060204^T^ which to the best of our knowledge is the first genome sequence of the species *M. kyorinense.*

### Nucleotide sequence accession numbers.

The whole genome sequence of KUM060204^T^ has been deposited in DDBJ/EMBL/GenBank under the accession numbers BBKA01000001 to BBKA01000453.

## References

[1] Okazaki M Ohkusu K Hata H Ohnishi H Sugahara K Kawamura C Fujiwara N Matsumoto S Nishiuchi Y Toyoda K Saito H Yonetani S Fukugawa Y Yamamoto M Wada H Sejimo A Ebina A Goto H Ezaki T Watanabe T. 2009. *Mycobacterium kyorinense* sp. nov., a novel, slow-growing species, related to *Mycobacterium celatum*, isolated from human clinical specimens. Int. J. Syst. Evol. Microbiol. 59:1336–1341. 10.1099/ijs.0.000760-0 19502312

[2] Ramos JP Campos CE Caldas PC Ferreira NV da Silva MV Redner P Campelo CL Vale SF Barroso EC Medeiros RF Montes FC Galvão TC Tortoli E. 2013. *Mycobacterium fragae* sp. nov., a non-chromogenic species isolated from human respiratory specimens. Int. J. Syst. Evol. Microbiol. 63:2583–2587. 10.1099/ijs.0.046862-0 23264503

[3] Campos CE Caldas PC Ohnishi H Watanabe T Ohtsuka K Matsushima S Ferreira NV da Silva MV Redner P de Carvalho LD Medeiros RF Abbud Filho JA Montes FC Galvão TC Ramos JP. 2012. First isolation of *Mycobacterium kyorinense* from clinical specimens in Brazil. J. Clin. Microbiol. 50:2477–2478. 10.1128/JCM.00023-12 22518856 PMC3405614

[4] Ohnishi H Yonetani S Matsushima S Wada H Takeshita K Kuramochi D Caldas PC Campos CE da Costa BP Ramos JP Mikura S Narisawa E Fujita A Funayama Y Kobashi Y Sakakibara Y Ishiyama Y Takakura S Goto H Watanabe T. 2013. *Mycobacterium kyorinense* infection. Emerg. Infect. Dis. 19:508–510. 10.3201/eid1903.120591 23750358 PMC3647647

[5] Telenti A Imboden P Marchesi F Lowrie D Cole S Colston MJ Matter L Schopfer K Bodmer T. 1993. Detection of rifampicin-resistance mutations in *Mycobacterium tuberculosis*. Lancet 341:647–650. 10.1016/0140-6736(93)90417-F 8095569

[6] Zhang Y Telenti Y. 2000. Genetics of drug resistance in *Mycobacterium tuberculosis*, p 235–254. *In* Hatfull GF Jacobs WR (ed), Molecular genetics of Mycobacteria. ASM Press, Washington, D.C.

